# Peptide VSAK maintains tissue glucose uptake and attenuates pro-inflammatory responses caused by LPS in an experimental model of the systemic inflammatory response syndrome: a PET study

**DOI:** 10.1038/s41598-021-94224-2

**Published:** 2021-07-20

**Authors:** Ismael Luna-Reyes, Eréndira G. Pérez-Hernández, Blanca Delgado-Coello, Miguel Ángel Ávila-Rodríguez, Jaime Mas-Oliva

**Affiliations:** 1 Instituto de Fisiología Celular, Universidad Nacional Autónoma de México, Circuito Exterior, Cd. Universitaria, 04510 Mexico, Mexico; 2 Facultad de Medicina, Universidad Nacional Autónoma de México, 04510 Mexico, Mexico

**Keywords:** Biochemistry, Drug discovery, Diseases, Molecular medicine, Pathogenesis

## Abstract

The present investigation using Positron Emission Tomography shows how peptide VSAK can reduce the detrimental effects produced by lipopolysaccharides in Dutch dwarf rabbits, used to develop the Systemic Inflammatory Response Syndrome (SIRS). Animals concomitantly treated with lipopolysaccharides (LPS) and peptide VSAK show important protection in the loss of radiolabeled-glucose uptake observed in diverse organs when animals are exclusively treated with LPS. Treatment with peptide VSAK prevented the onset of changes in serum levels of glucose and insulin associated with the establishment of SIRS and the insulin resistance-like syndrome. Treatment with peptide VSAK also allowed an important attenuation in the circulating levels of pro-inflammatory molecules in LPS-treated animals. As a whole, our data suggest that peptide VSAK might be considered as a candidate in the development of new therapeutic possibilities focused on mitigating the harmful effects produced by lipopolysaccharides during the course of SIRS.

## Introduction

During the last years, infections have re-emerged as one of the most important public health problems around the globe. Since nowadays the aggravated states of an infectious process, such as sepsis and septic shock are the main causes of death in Intensive Care Units worldwide, there is the imperative need to find new ways not only to diagnose but also to successfully treat these conditions^1,2^. Sepsis is characterized by the presence of infection in association with a dysregulated host response and organ failure; while, septic shock also includes the development of systemic hypotension^3,4^. Dysregulation of this response is mainly characterized by an imbalance in the systems that regulate the host's immune activation when the presence of the pathogen leads to the development of a hyper-inflammatory state followed by the exhaustion of the immune system to adequately respond^5^. With this concept in mind, and the goal to establish a set of guidelines that would allow to study the translational value of preclinical experimental models to the clinical setting, it is now established that the term Systemic Inflammatory Response Syndrome (SIRS), corresponds to a more appropriate term than experimental sepsis when LPS are used to trigger an inflammatory response^6^.

The recognition of molecules by the innate immune system has a leading role in the host's response through molecules present in most pathogenic organisms that stimulate the development of a systemic defense. As a whole, such molecules known as MAMPS for Microorganism Associated Molecular Patterns^7^, can be recognized through receptors that collectively are known as Pattern Recognition Receptors or PRR's. Toll-like receptors (TLRs) are considered one of the main families of receptors included among PRR's. Within the TLRs, the type 4 receptor (TLR4) plays an important role in the development of the host immune response during bacterial infection^8^ through the recognition of lipopolysaccharides (LPS), one of the most immunogenic MAMPS and responsible for the development of septic shock in infections caused by Gram-negative bacteria^9–11^.

LPS molecules present three well-defined regions within their structure (lipid A, a core region and the O antigen), that show a large degree of variability independent of each other^12^. Lipid A considered the most conserved region of LPS, presents special importance for recognition by the immune system. When this region binds to a member of the lipopolysaccharide-binding protein family, the complex is recognized by the TLR4 receptor, and the development of the host response initiates^13^.

Lipopolysaccharide-binding proteins are part of a family of proteins known as PLUNC-containing-motif proteins (PLUNC for Palate, Lung, Nasal-epithelium Clone-Protein). PLUNC proteins are involved in the defense system against pathogens of the upper airways and palate^14–16^. Sharing a common structural motif, so far this family includes four members: the bactericidal permeability-increasing protein (BPI); the lipopolysaccharide-binding protein (LBP); the phospholipid-transfer protein (PLTP), the cholesterol-ester transfer protein (CETP), and the cholesterol-ester transfer protein intestinal isoform (CETPI) discovered by our group^17–19^.

CETP corresponds to a plasma protein involved in the transfer of triacylglycerols and cholesterol-esters between lipoproteins, showing a critical role in the homeostasis of cholesterol metabolism and therefore being responsible for the reverse transport of cholesterol^20–22^. Several years ago, studying this protein, our group discovered the presence in the human plasma of a new variant of CETP, called at that time cholesterol-ester transfer protein isoform from the intestine or CETPI^17^. Since then, we have reported that CETPI shares a very high degree of structural conservation with respect to CETP (~ 96%) while maintaining the same secondary and tertiary structures. The only difference found between these proteins resides in a change in the last 23 amino acids of the carboxy-end segment of CETP. Interestingly, despite being almost identical, this minimal difference allows both proteins to perform completely different functions^23^.

By using a series of synthetic peptides derived from the carboxy-end segment of CETPI, we have established an important LPS binding property for this new protein. Therefore, employing a synthetic peptide containing 18 amino acids derived from this carboxy-end segment of CETPI (named VSAK for its first amino-end four amino acids), we have described that the interaction between peptide VSAK and LPS can neutralize LPS induced toxicity in vitro as well as in vivo. Moreover, the in vivo administration of peptide VSAK in an experimental animal model of septic shock, demonstrated that the intravenous administration of this peptide prevents the harmful effects of LPS associated with the development of this acute clinical condition^18^. As an extension of these results, the present investigation considered as proof of concept has allowed us to further test the in vivo action of peptide VSAK in LPS treated experimental animals using Positron Emission Tomography (PET), by measuring glucose metabolism through 2-deoxy-2-[^18^F]fluoro-D-glucose ([^18^F]FDG) tissue uptake in parallel with the study of pro-inflammatory cytokines, plasma glucose, and insulin. [^18^F]FDG corresponds to a radiolabeled glucose analogue that is actively incorporated into the cell through glucose transporters (GLUTs), then phosphorylated by hexokinases to FDG-6-phosphate, and trapped within the cell. The uptake of this radiotracer reflects tissue glucose uptake and metabolism.

## Results

### Peptide VSAK prevents the deleterious effects of circulating LPS allowing a normal tissue [^18^F]FDG permeation in a model of the systemic inflammatory response syndrome

**Figure 1:**
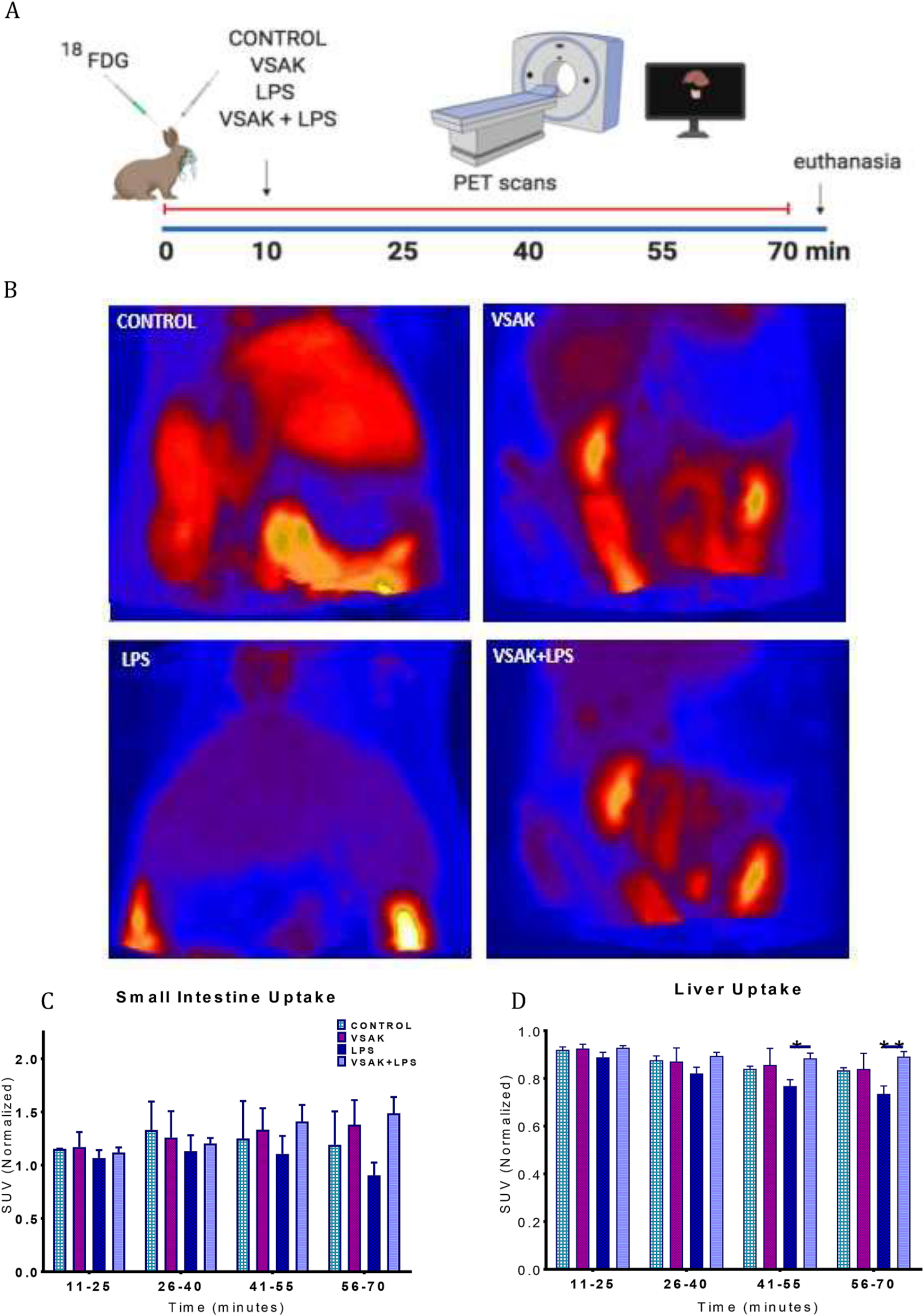
Systemic [^18^F]FDG uptake after the administration of LPS (300 ng/kg) and/or peptide VSAK (60 µg/kg). (**A**) Schematic representation showing the administration of treatments and PET acquisition procedures. (**B**) PET images showing the global [^18^F]FDG uptake of representative experimental rabbits during a 70 min acquisition time. Quantitative analysis for the small intestine (**C**) and the liver (**D**) during the different time intervals of PET acquisition (mean of normalized SUV ± SEM). Statistical differences were found in the liver between the LPS and LPS + VSAK groups at 55 min (*p* = 0.027 *) and 70 min (*p* = 0.002 **). Control, VSAK; LPS and VSAK + LPS groups, n = 3 for each group. Differences assessed using two-way ANOVA with Tukey’s multiple comparison tests.

Images were generated from data obtained during the PET experiment by using a dynamic scan data acquisition format (Fig. 1A). These images correspond to total [^18^F]FDG uptake observed at the end of the experiment (minute 70) in a representative animal from each experimental group (Fig. 1B, Supplementary Videos S1–S4)). Control groups consist of animals injected only [^18^F]FDG, or peptide VSAK (Fig. 1B, Supplementary Videos S1–S2). Consistently with the three animals studied, an important decrease in [^18^F]FDG uptake was observed in the LPS treated group with respect to controls (Fig. 1B, Supplementary Video S3). Remarkably, animals injected in the opposite ears within a 1-min interval with LPS and peptide VSAK, present [^18^F]FDG uptake values qualitatively similar to images seen in the control groups, demonstrating the LPS buffering capability of peptide VSAK (Fig. 1B, Supplementary Video S4).

Quantitative analysis performed on intestine and liver tissues from this set of animals is shown in Fig. 1C,D. Standard Uptake Values (SUV) for these tissues were normalized using the first 10 min as basal uptake. Related to the number of samples measured, although no statistical differences were found among experimental groups when SUV for [^18^F]FDG is measured in the intestine, there is an interesting trend showing an attenuation from the LPS effects carried out by peptide VSAK (Fig. 1C), results that agree with the PET images shown earlier.

When SUV for [^18^F]FDG from animals treated under the same referred experimental conditions was measured in the liver at 25 and 40 min after the start of the experiment, there are no significant differences in [^18^F]FDG uptake (Fig. 1D). Nevertheless, after 55 min had elapsed, a significant recovery in [^18^F]FDG uptake was found between the LPS and the VSAK + LPS group. These differences, maintained until the end of the experiment (70 min), again, are consistent with the PET images shown in Fig. 1B.

Since this experiment did not seem to present clear differences between experimental groups during the time frames evaluated, including the first 10 min of basal PET data acquisition, a second experiment was designed applying the administration of [^18^F]FDG and treatments at time zero, increasing the LPS concentration used, and the PET acquisition time from 70 to 90 min.

**Figure 2:**
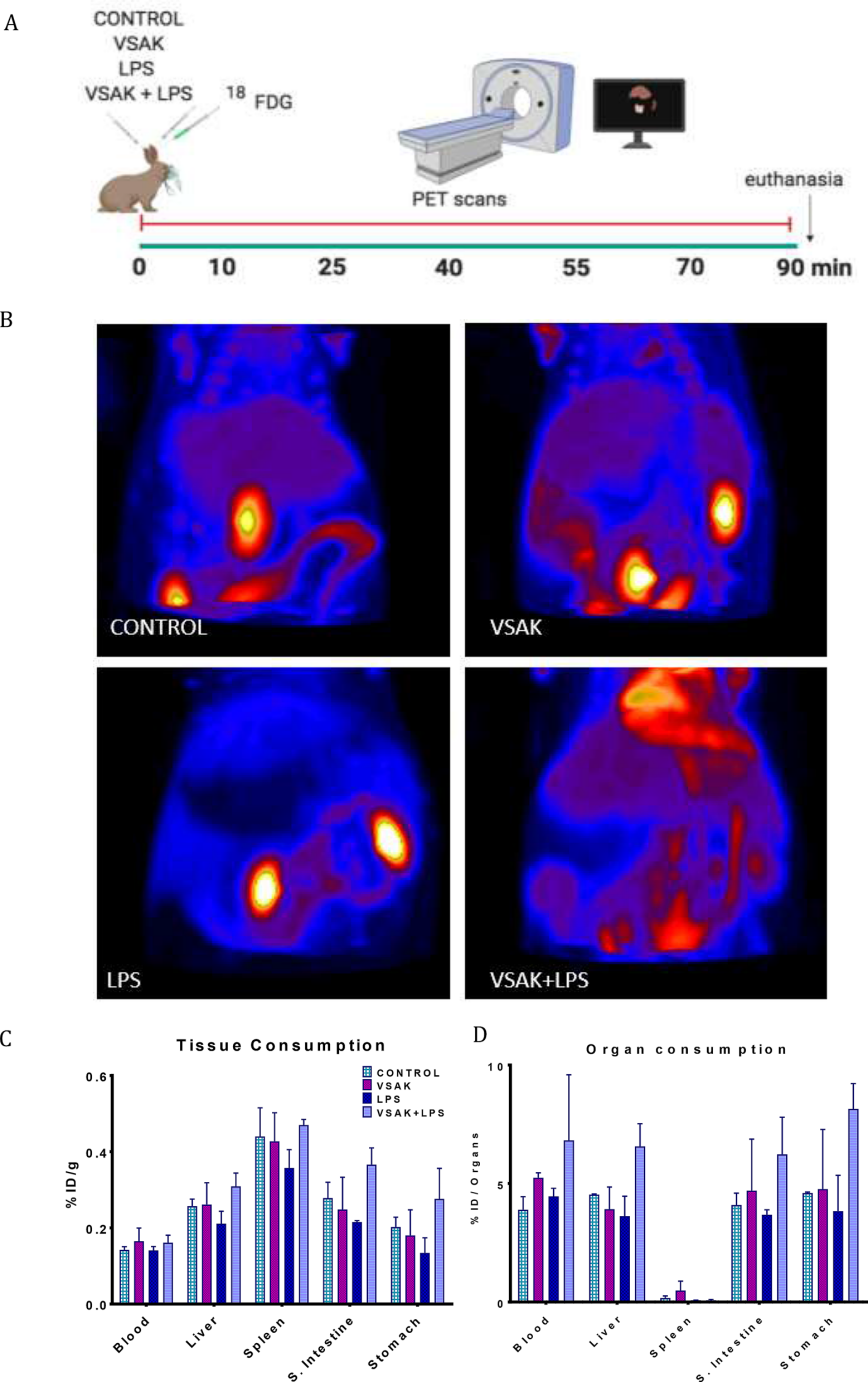
Systemic [^18^F]FDG uptake after the administration of LPS (450 ng/kg) and/or peptide VSAK (60 µg/kg). (**A**) Schematic representation showing the administration of treatments and PET acquisition procedures. (**B**) PET images showing the global ^18^FDG uptake of representative experimental rabbits during the 90-min acquisition time. (**C**) The quantitative analysis expressed as a percentage of the injected dose per gram of tissue (%ID/g). (**D**) percentage of injected dose per organ (%ID/organ). Control, VSAK; LPS and VSAK + LPS groups, n = 3. Differences assessed using two-way ANOVA with Tukey’s multiple comparison tests.

This new set of experiments employing a higher LPS dose (450 ng/kg) was conducted to evaluate the potential neutralizing effect of peptide VSAK under LPS saturating conditions (Fig. 2A). PET images from this experiment are shown in Fig. 2B, and supplementary videos S5–S8. In accordance to results obtained during the first experiment, a similar response can be observed, where tissue [^18^F]FDG perfusion dramatically drops when a higher LPS concentration is used (Fig. 2B, Supplementary Video S7). This decrease in glucose perfusion is countered with the intravenous injection of peptide VSAK (Fig. 2B, Supplementary Video S8).

**Figure 3:**
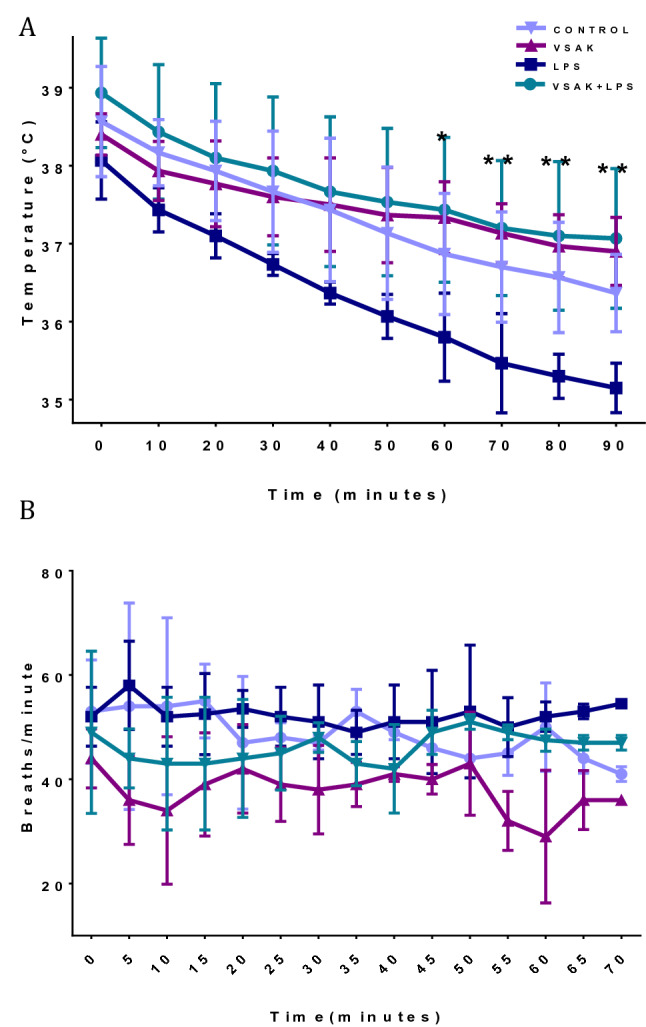
Vital signs of experimental animals under treatment with LPS (450 ng/kg) and/or peptide VSAK (60 µg/kg) registered throughout the PET study. (**A**) Temperature recordings were obtained every 5 min during the 90-min duration of the experiment. At 60 min significant differences started to be seen: LPS / VSAK + LPS groups (*p = 0.03). Between 70–90 min: LPS / VSAK groups (**p = 0.007); LPS / VSAK + LPS groups (**p = 0.003). Control, VSAK; LPS and LPS + VSAK groups, n = 3. (**B**) Respiratory frequency (n = 3). Differences assessed using two-way ANOVA with Tukey’s multiple comparison tests.

As shown in Fig. 2C, a direct measurement of Specific Tissue Activity (%ID/tissue) and Specific Organ Consumption (%ID/organ) from the same set of experimental animals was carried out in whole organs and samples from blood, liver, spleen, small intestine, and stomach (Fig. 2C,D). Even though due to the number of animals studied, no statistical significance was found among groups in both types of analysis, there is a clear tendency for LPS to decrease [^18^F]FDG uptake, a situation that is reversed by the infusion of peptide VSAK. Again, there seems to be a protective trend against the action of LPS caused by the presence of peptide VSAK in circulation. Also, body temperature and respiratory frequency of animals from the different experimental groups were measured along with the PET experiments (Fig. 3). Although most probably due to the effect of the anesthetic, a continuous decrease in temperature could be observed in all animals from the different groups, there is a tendency for body temperature to decrease in all groups, and specially in the LPS group. At the end of the experiments, this last group showed severe hypothermia, a situation that was prevented when experimental animals were treated with peptide VSAK (Fig. 3A). These results indicate the ability of peptide VSAK to attenuate the continuous drop in temperature associated with hypothermia during the establishment of SIRS. On the other hand, since the parameter of respiratory frequency was employed to adjust the dosage of anesthetic and as an indicator of the effect of anesthesia during the procedure, there are no apparent changes between groups. Nevertheless, the group of animals treated with LPS and peptide VSAK seemed to present lower respiratory frequency values in comparison to the other groups (Fig. 3B).

Since a limitation of our study might be the number of experimental animals employed, it has to be mentioned that although a series of preliminary experiments were performed, these experiments that were not included in the study we report, showed the same protective role exerted by peptide VSAK upon LPS derived SIRS.

### Peptide VSAK prevents an increase in glucose reuptake by the kidney

**Figure 4:**
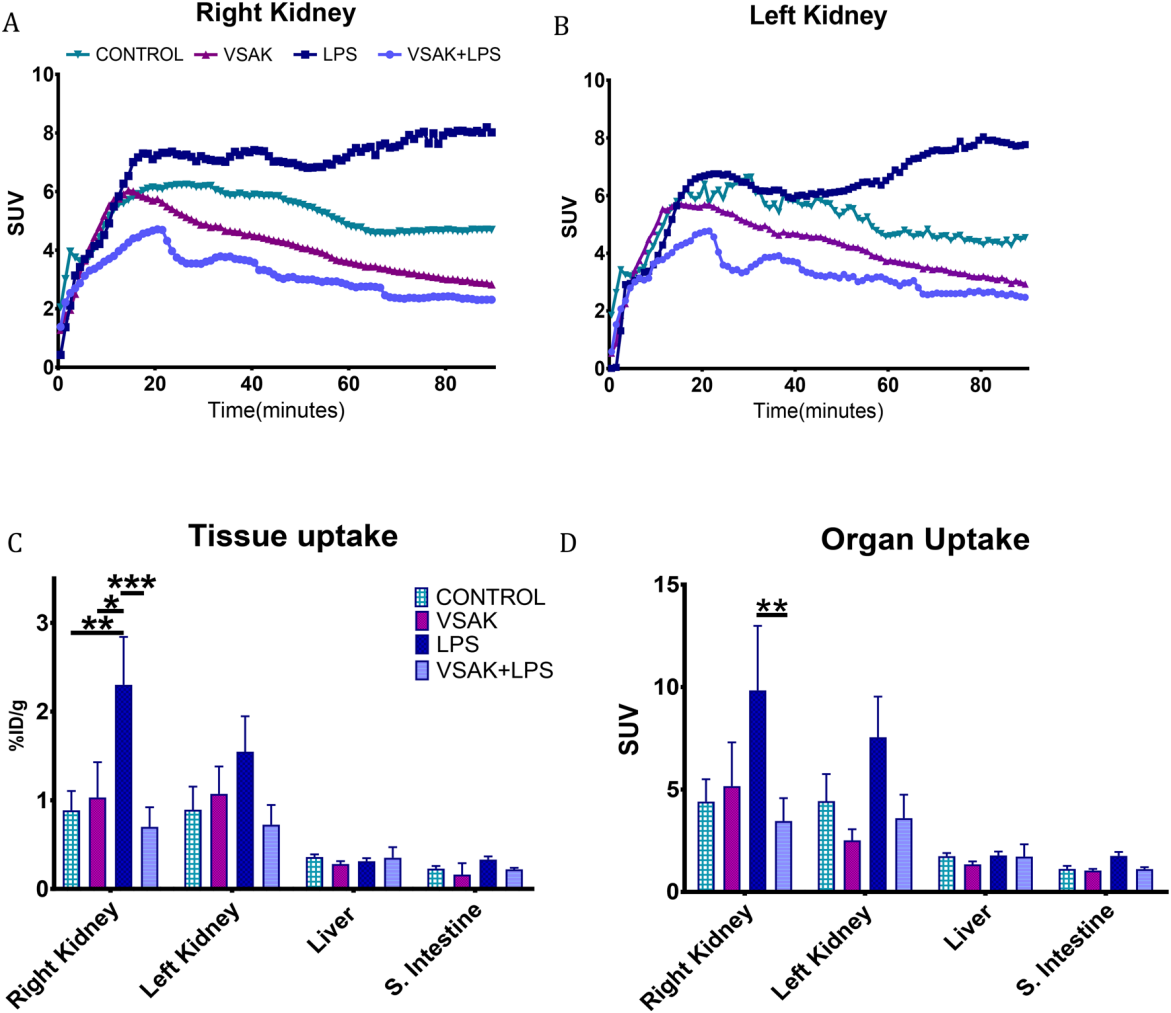
SUV analysis of organ throughout the experiment using 1-min frames. (**A**) Right kidney. LPS (7.33 ± 3.09); LPS + VSAK groups (2.77 ± 0.64) at minute 60 (*p = 0.04). This difference was maintained during the 90 min of the experiment. VSAK (2.82 ± 1.42); LPS (8.02 ± 4.61) (*p = 0.04); LPS + VSAK (2.3 ± 0.76) (**p = 0.008). (**B**) Left kidney. (**C**) Percentage comparison of injected doses (%ID/g) observed in the kidney, liver, and small intestine. Right kidney: (**p = 0.005); (*p = 0.013); (***p = 0.0005). Although a similar behavior was observed in the left kidney, non-significant differences were found. (**D**) SUV analysis. Right kidney; (*p = 0.0085). (n = 3 in all groups studied). Differences assessed using two-way ANOVA with Tukey’s multiple comparison tests.

Changes in glucose renal reuptake were analyzed with data obtained from the kidneys of animals in all experimental groups (Fig. 4A,B). Although during the analysis of data throughout the 90-min duration of the PET experiments, we found slight differences in reuptake between the right and left kidneys, the group including LPS treated animals showed the highest value of kidney glucose reuptake. In contrast, over time, control groups show that renal glucose reuptake tends to decrease. Interestingly, the LPS/peptide VSAK group showed the lowest reuptake values. This result is most probably related to the fact that this group of animals presented a normal tissue glucose uptake, very much similar to what it is found with control groups.

To corroborate the effect observed in SUV through time, total values from both kidneys were studied in parallel to the liver and intestine (Fig. 4C,D). This analysis was performed using data acquisition parameters to obtain total %ID/cc and SUV from each organ. Following the previous analysis, we observed that the highest values for these parameters calculated in both kidneys correspond to the LPS treated group. Moreover, the protective effect carried out by the administration of peptide VSAK concomitantly with LPS is kept in all tissues studied. In concordance to the PET images presented earlier, the liver and the intestine showed much lower SUV and %ID/cc values than kidneys.

### Peptide VSAK reduces the level of pro-inflammatory molecules in circulation

**Figure 5:**
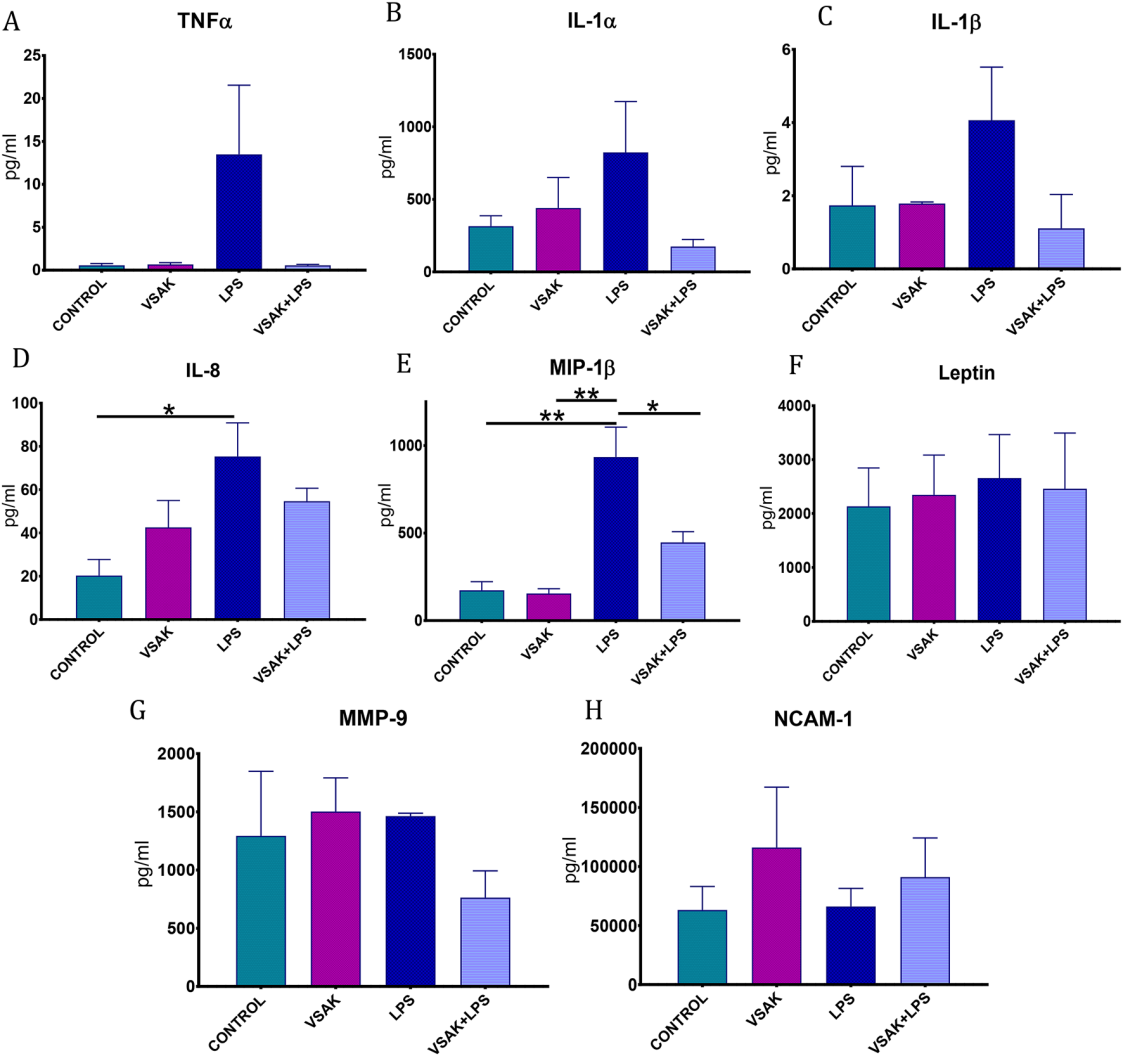
Quantitative estimation of pro-inflammatory cytokines in the plasma of rabbits treated with LPS (450 ng/kg). (**A**) TNFα. (**B**) IL-1α. (**C**) IL-1β. (**D**) IL-8. (*p = 0.03). (**E**) MIP-1β. Control / LPS groups (**p = 0.002), LPS / LPS + VSAK groups (*p = 0.027). (**F**) Leptin. (**G**) MMP-9. (**H**) NCAM-1. Control, VSAK; LPS and VSAK + LPS groups, n = 3. Differences assessed using one-way ANOVA with Tukey’s multiple comparison tests.

The level of a series of circulating cytokines investigated in all experimental groups directly associated with the pro-inflammatory response during the process of sepsis and septic shock are shown in Fig. 5. Studying the same animal groups employed for the PET analysis, we observed that while control groups show low levels of TNFα, IL-1α, IL-1β, IL-8, and MIP-1β, the group of animals treated with LPS importantly increases the level of these cytokines. Interestingly, this response is prevented when peptide VSAK is in parallel infused with LPS.

When the circulating levels of Leptin, MMP-9, and NCAM-1 were measured in the different groups of animals, no differences among groups were observed. Nevertheless, the level of TNF-α that dramatically increases in the LPS treated group, is completely prevented by the intravenous infusion of peptide VSAK, as consistently observed by us^18^.

**Figure 6:**
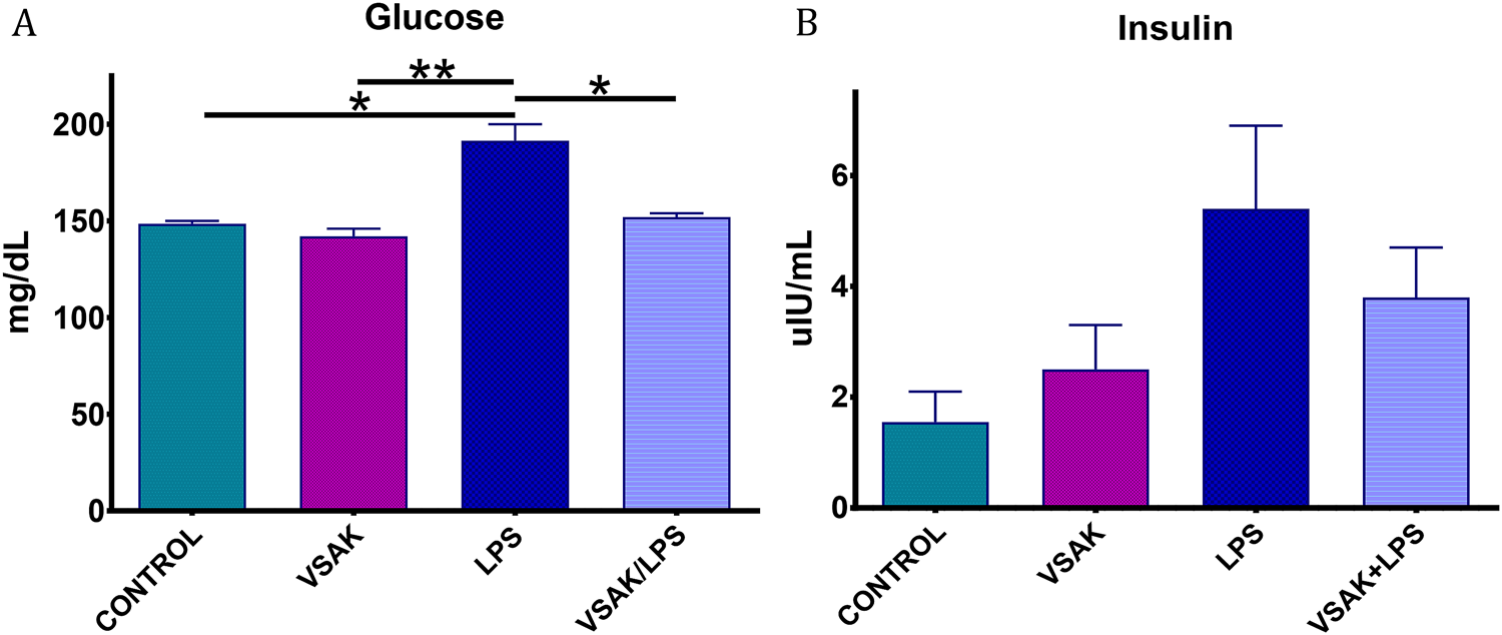
Plasma glucose and insulin levels from experimental rabbits after the administration of LPS (450 ng/kg). (**A**) Glucose level. (**p = 0.006); (*p = 0.015). (**B**) Insulin level. (n = 3 in all groups studied). Differences assessed using one-way ANOVA with Tukey’s multiple comparison tests.

To further correlate the observation made by PET associating the neutralization of LPS carried out by peptide VSAK and the lowering effect in the levels of circulating cytokines, we also analyzed the levels of circulating glucose and insulin in association with the effects produced by LPS and LPS/peptide VSAK administration (Fig. 6). It can be appreciated that the level of glucose in the LPS group is significantly higher than that observed in the control groups (Fig. 6A). Again, the infusion of peptide VSAK in parallel with LPS allows glucose to be kept at levels similar to the ones measured in control groups. A similar correlation is found when insulin is studied, finding a tendency for insulin to increase in the LPS group and recovery close to control values observed after peptide VSAK treatment (Fig. 6B). Interestingly, despite the high level of insulin shown in animals from the LPS group, these experimental animals show the highest level of circulating glucose, a phenomenon that is prevented by the infusion of peptide VSAK.

### Molecular dynamics

**Figure 7:**
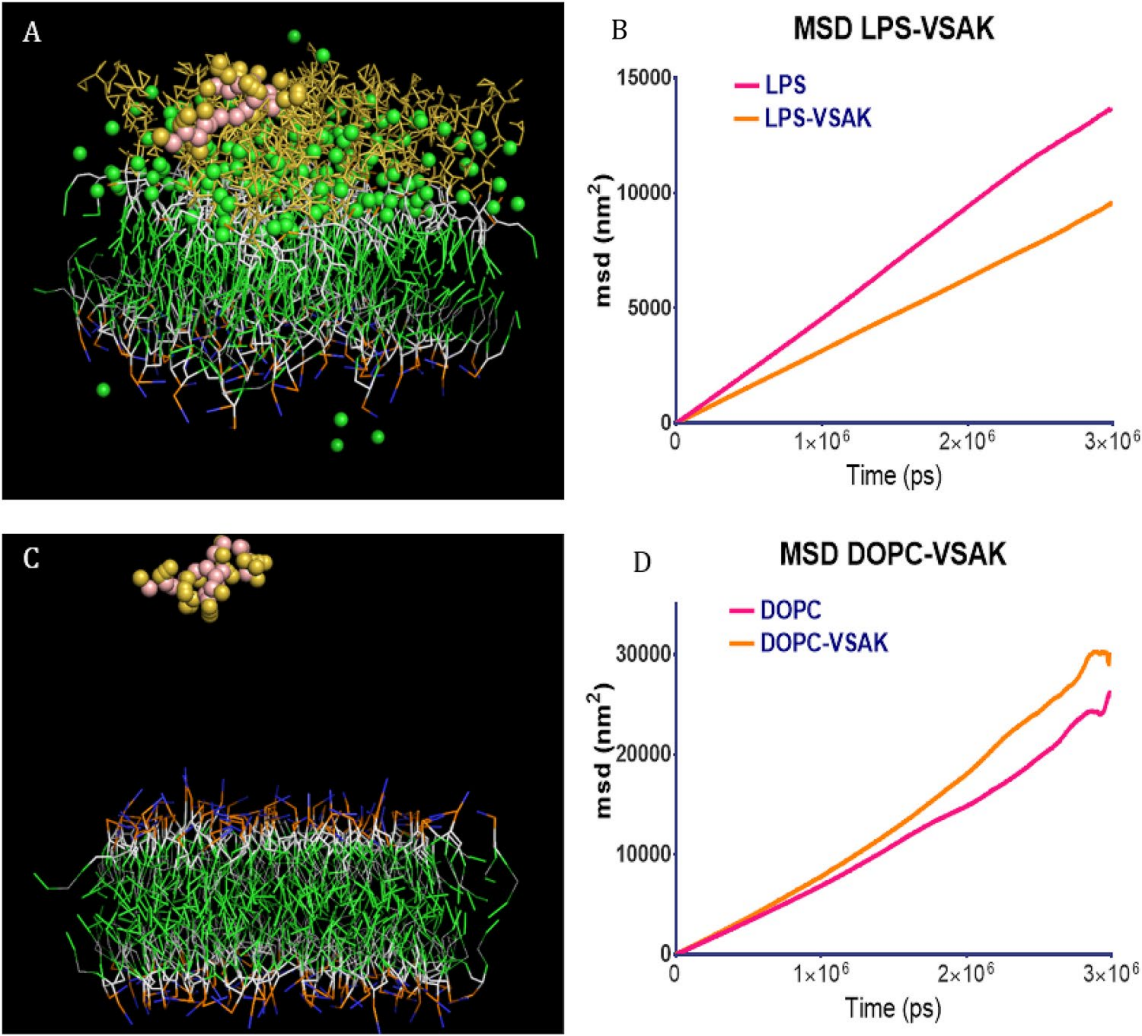
Molecular dynamics simulations employing lipid bilayers composed of LPS and/or DOPC. (**A**) Snapshot of a bilayer at 2250 ns containing LPS/DOPC interacting with peptide VSAK. (**B**) Mean square displacement (MSD) analysis of the LPS/DOPC system in the presence or absence of peptide VSAK. (**C**) Snapshot at 2250 ns of a bilayer made entirely of DOPC in the presence of peptide VSAK. (**D**) MSD values from the DOPC system in the presence or absence of peptide VSAK.

The use of molecular dynamics has allowed us to start to understand the type of interactions that might be occurring in vivo between peptide VSAK and LPS. Figure 7A shows a captured frame of a dynamic system consisting of a bilayer whose external hemi-layer is made up of LPS, while the internal one is made up of DOPC. A simulation image frame obtained at 2250 ns shows peptide VSAK positioned in contact with the external hemi-layer of the bilayer, showing a slight bilayer penetration towards the core area of LPS. During this simulation, it is observed that once peptide VSAK reaches the bilayer, it remains close to this region restricting its lateral movement (Fig. 7B, Supplementary Video S9). A mean square displacement (MSD) analysis for this set of simulations using a LPS/DOPC composite system shows the center of mass placed on the membrane, while the total movement in the lateral plane (z) analyzed as an indicator of a change in membrane fluidity is decreased by the presence of the peptide (Fig. 7B). Furthermore, to analyze whether this effect is directly related to the presence of LPS, bilayer systems consisting exclusively of DOPC were generated. Figure 7C shows a snapshot frame of one of these systems captured at 2250 ns corresponding to a simulation containing a membrane bilayer system composed of DOPC and no LPS in the presence of peptide VSAK. It is interesting to observe that MSD values are not affected when peptide VSAK apparently does not interact with the membrane (Fig. 7D, Supplementary Video S10).

## Discussion

The use of functional imaging techniques such as PET nowadays has had a limited impact on the study of clinical conditions such as infection, sepsis, and septic shock. In many cases, the use of this technique has been limited as a diagnostic support tool when the source of the infection does not have a known origin. In basic research, the use of this technique for the study of these pathologies has also been quite limited and in a similar way to the clinical field, used mainly to study the development of infectious processes in situ^24,25^.

During the present study, we continued with our strategy to use an experimental animal model closer to the human^26–28^. Although in the past our research group used the New Zealand breed of rabbits to establish the preventive actions of peptide VSAK against the deleterious effects of LPS in experimental septic shock^18^, the present study has been carried out employing Dutch dwarf rabbits, that in the adult stage have a similar size to that of an adult Wistar rat. This strategy has permitted us to employ this animal model in a piece of micro PET equipment, originally designed to study mice and rats.

The most noticeable effect found in the experimental group of animals intravenously injected with LPS was the almost total absence of glucose uptake shown by most organs in two independent sets of PET experiments, an observation supported by the quantitative analysis of SUV data and %ID/cc values. Although most differences found to represent a relatively small change in the total capture of radiolabeled-glucose, our results still represent an important event in the context of the whole organism, something common when considering that one of the advantages of functional imaging techniques is to highlight very small variations in the specific region of study. This point is observed after the administration of LPS, associated with an important increase in glucose uptake by the kidneys. This effect became more noticeable as capture time advances reflected in the total uptake data for this organ. Nevertheless, the most dramatic effect found in our study is associated with the group simultaneously treated with LPS and peptide VSAK, where our experimental animals showed normal organ perfusion values and did not present any of the clinical signs associated with SIRS.

The decrease in organ glucose uptake due to the administration of LPS countered by the administration of peptide VSAK is not observed in the kidneys, where because of the administration of LPS an increase in glucose recapture was found. Since it has been proposed this late phenomenon is not associated with a change in the metabolic activity of the kidney^29^, it can be related to the fact there is an increased recapture of glucose when most organs have become refractory to this sugar. All the changes observed in the uptake of radiotracer upon the administration of LPS, indicate there is an alteration in the mechanisms that regulate the entry of glucose into the cells. This alteration was confirmed by analyzing the circulating levels of glucose and insulin. While a slight increase in insulin associated with an important increase in the circulating level of glucose was observed, a phenomenon frequently related to metabolic dysfunction, the administration of peptide VSAK attenuated the increase of both parameters. Therefore, it is proposed that treatment with peptide VSAK might also prevent the establishment of an insulin-resistance-like condition^30–32^.

This effect is consistent with observations from the clinical setting, where a transient appearance of insulin resistance is a characteristic event of sepsis and septic shock^33^, and where the appearance of hyperinsulinemia and hyperglycemia associated with metabolic dysfunction have been considered as a negative indicator for the prognosis of sepsis and septic shock^34^. During these clinical conditions, the establishment of an insulin resistance state is associated with the production of pro-inflammatory molecules, whose effects are known to interfere with the normal signaling pathway for insulin^35–37^. Moreover, the appearance of hypothermia that regularly occurs during the critical phases of sepsis and septic shock^38^ known to be exacerbated by the use of anesthetics^39^, can be also related to the progressive decrease in tissue glucose uptake and the alteration of the cellular intermediate metabolism involved in the production of heat^40^. Interestingly, independently of the origin of this harmful clinical sign, the administration of peptide VSAK preventing the onset of hypothermia maintains organs within their normal metabolic homeostasis.

Since there is evidence that hyperglycemia observed during the course of sepsis and septic shock, can be due in part to the inability for glucose to enter the cell, energy deficiency has been proposed among the mechanisms responsible for cell dysfunction in septic shock, where low ATP levels and phosphocreatine/ATP ratios have been associated to non-surviving cases of septic shock^41,42^. This is supported by clinical studies where, for example, serum glucose measured in children presenting signs of septic shock and high plasma glucose levels, are associated to high mortality rates^43^. By studying tissue [^18^F]FDG incorporation, our study supports the fact that by improving tissue glucose uptake, and in parallel reducing hyperglycemia close to normal levels as achieved here employing peptide VSAK, the chances for survival dramatically improve.

During the course of our study, the analysis of a series of pro-inflammatory molecules known to be involved in the pathophysiology of sepsis and septic shock were also studied. These included TNFα, IL-1α, IL-1β, IL-8, MIP-1β, Leptin, soluble NCAM-1, and MMP-9. Among them, TNFα, IL-1α, IL-1 β are known to intervene in the development of an acute inflammatory response and therefore associated with the early development of SIRS, while chemokines MIP-1β and IL-8 are produced in an intermediate phase of septic shock and associated with the migration of cells from the immune system to sites of infection to promote inflammation. TNFα that presents a central role in the response that leads to sepsis and septic shock, has been directly involved in the host's response to LPS mediated by the TLR4 receptor^44^, and also associated to the development of insulin resistance^45–47^.

The analysis of these pro-inflammatory markers was completed with Leptin, MMP-9, and NCAM-1. Unlike cytokines and chemokines, these molecules are associated with the later stages in the development of the inflammatory process. Since the analysis of these last three molecules showed no significant changes among the various groups of experimental animals studied, it can be considered that our model of SIRS corresponds to an acute model where the late phases of sepsis and septic shock are still not reached^48^.

Interestingly, except for these three molecules, TNFα, IL-1α, IL-1β, IL-8, and MIP-1β showed an important increase in their circulation level after the infusion of LPS, a response that is avoided if peptide VSAK is concomitantly administered. As shown before by us, TNFα one of the pro-inflammatory cytokines that importantly increase with LPS treatment is kept at basal control levels if peptide VSAK is employed, supporting the view that attenuation of the various pro-inflammatory molecules carried out by peptide VSAK occurs during the early stages of the inflammatory process. The potential beneficial effects of peptide VSAK during the late stages of SIRS will have to be further studied.

On the other hand, to explain not only the in vitro but the in vivo effects observed after the administration of peptide VSAK, we believe it is important to understand first the type of interactions that may occur between peptide VSAK and LPS. Therefore, in this study, we also decided to tackle this phenomenon through the use of molecular dynamics. Coarse grain molecular dynamics of VSAK peptide studied in association with LPS/DOPC composite systems shows there is a decrease of peptide displacement in the lateral plane of the membrane (MSD) representative of the membrane fluidity, in contrast to the normal displacement value when peptide VSAK interacts with a membrane composed only of DOPC. This result supports the possibility that interactions between peptide and LPS already located in a membrane, might be able to decrease membrane fluidity that in turn could interfere with the harmful cascade of cellular events known to be carried out by LPS^49,50^.

The modulating effect of peptide VSAK showed in this study acquires special importance when considering that several of the experimental treatments investigated and designed to counteract sepsis and septic shock have failed during their study in clinical phases due mainly to the presence of immunosuppression^51–54^. The LPS-neutralizing effect shown by peptide VSAK raises the possibility for the use of this peptide as a therapeutic agent in conjunction with the standard treatment approaches during the acute stages of SIRS in seriously ill patients at the intensive care unit^55^^.^^56^.

## Materials and methods

### Study design

Based on the fact that VSAK peptide can bind LPS, the goal of the present study was to find additional evidence concerning the possibility that the intravenous administration of peptide VSAK results in a protective measure for animals exposed to LPS in a model of SIRS. In this study, we used as an experimental model, Dutch dwarf rabbits, found by us to be an appropriate model to evaluate the systemic effects of the administration of LPS employing micro-PET imaging. Since our investigation has been designed as an end-point study, fluid resuscitation employing iso-osmolar crystalloid solutions which is a standard in patient management, was not carried out. Also, as a limitation of the study, it has to be mentioned that taking into account the duration of each experiment, and the fact that LPS are administered almost at the same time with peptide VSAK, our model is not 100% reflective of a clinical situation.

### Reagents

Purified LPS from *Escherichia coli* O111: B4 was purchased from Sigma-Aldrich (St. Louis, MO, USA; Cat. L2630). LPS were solubilized at a final concentration of 10 mg/ml in glucose-free Krebs–Ringer buffer (125 mM NaCl, 2.5 mM KCl, 1.25 mM NaH2PO4, 2 mM CaCl2, 1 mM MgCl2, 25 mM NaHCO3), at pH 7.4. VSAK peptide (VSAKPLSARSPGGRPLSP) was synthesized by GenScript USA Inc., (Piscataway, NJ, USA) with a purity of 98%. Peptide VSAK was diluted in PBS buffer at a final concentration of 1 mg/ml. The radiotracer 2-[18]-fluoro-2-deoxy-D-glucose ([^18^F]FDG) was synthesized and provided by the Radiopharmacy-Cyclotron Unit located at Facultad de Medicina, Universidad Nacional Autónoma de México.

### Experimental animals

All procedures with experimental animals were performed following the Guide for Care and Use of Laboratory Animals from the NIH, and the current Mexican Official Norm for the Use of Laboratory Animals (NOM-062-ZOO-199). All experimental protocols were revised and approved by the Animal Care and Use Committee of Instituto de Fisiología Celular, Universidad Nacional Autónoma de México (Protocol JMO2017-17).

Twenty-four male Dutch dwarf rabbits (45 days old) were purchased from a local certified farm. Rabbits were maintained in a quarantine period for 15 days under controlled conditions of temperature, humidity, light–dark cycles, and ad libitum access to food and water. Before the microPET assays, animals fasted for 12 h and were weighed 1 h before the start of the experiments to precisely estimate the corresponding doses of the radiotracer. All procedures were performed under gaseous anesthesia (isoflurane) administered through a breathing mask along with oxygen. For anesthesia induction, rabbits were placed in a closed chamber and administered with 5% isoflurane. The dose of anesthetic was adjusted using the respiratory rate that was kept at 50–60 breaths per minute and maintained with 1–2.5% isoflurane during the scan acquisitions. To prevent a drastic temperature drop caused by the anesthetic, animals were covered with a thermal coat during PET scans.

### Experimental procedures

Rabbits were randomly assigned to four different experimental groups: Control, VSAK treatment, LPS treatment, and VSAK + LPS treatment. All treatments were administered via the marginal veins of the ears. For the first experiment, the control group was administered only with a saline solution, and the VSAK/control group administered 60 µg/kg of the peptide, as previously reported^18^. Animals from the LPS group were administered with 300 ng/kg LPS, and the VSAK + LPS group administered with 60 µg/kg of peptide VSAK and 300 ng/kg LPS. For the first experiment, animals were first administered with [^18^F]FDG and 10 min later received the corresponding treatment. For the second experiment, doses for control and VSAK groups were administered as followed for the first experiment, whereas the LPS doses were adjusted to 450 ng/kg. For this experiment, animals were simultaneously administered with peptide VSAK and LPS on different ears. Once the animals received the treatments, they were administered [^18^F]FDG and microPET images acquired.

### MicroPET equipment

The equipment employed for this study corresponds to a third-generation microPET Focus 120 Scanner (CTI-Concorde Microsystems LLC, Knoxville, TN, USA), designed for small animals. The microPET scanner is provided with a detection ring of 15 cm diameter with an array of 96 detector blocks, each of them consisting of a 12 × 12 array of lutetium oxyorthosilicate (LSO) crystals coupled to position-sensitive photomultiplier tubes. The equipment provides a field of view (FOV) of 7.6 cm by 11 cm in axial and transversal directions, respectively.

### Data acquisition

Before [^18^F]FDG administration, anesthetized experimental animals were placed in the scanner bed and the FOV centered in the area between the apex of the heart and the topside of the bladder. With this alignment, the liver, kidneys, and part of the small intestine can be studied. After the radiotracer is intravenously administered, dynamic PET data starts in list mode.

For the first set of experiments, PET data were acquired for 70 min considering the administration of [^18^F]FDG as zero time. After this time had elapsed, a blood sample was obtained by cardiac puncture and animals euthanized with an overdose of pentobarbital. For the second experiment employing 450 ng/kg LPS, PET data were acquired for 90 min after the administration of [^18^F]FDG. At the end of both experiments, blood samples were collected as well as samples from the liver, small intestine, spleen, and kidneys. For the second experiment, collected organs were weighed to estimate the total uptake of the radiotracer. All tissue samples were stored in liquid nitrogen.

### Image reconstruction and data analysis

PET images were reconstructed in a 3D mode with the ordered subset expectation maximization algorithm OSEM3D. Data analysis was carried out using spherical areas of 5 mm diameter to define the volume of interest (VOI) and to get the standardized uptake value (SUV) of the indicated organ. The 70 min scan acquisition data were analyzed in five frames: the first one comprising the initial 10 min (0–10 min), followed by four frames of 15 min each (10–25, 25–40, 40–55, and 55–70 min). To determine the percentage-injected dose per gram of tissue (%ID/g), density values of 1 g/cm3 were considered for all organs. Data from the first set of experiments were normalized with the SUV corresponding to the frame 0 to 10 min for each animal. To obtain the SUV of the second set of experiments, the data corresponding to 90 min of observation, were analyzed in 1-min frames. SUV estimations were obtained from the VOI data corresponding to the 90 min acquisition time. Image quantification was performed by the same person who acquired the images.

### Measurement of pro-inflammatory cytokines

The antibody rabbit cytokine array 1 from Raybiotech (Norcross, GA, USA; Cat. QAL-CYT-1) was used to carry out the specific measurement of ten pro-inflammatory cytokines in serum samples, according to the manufacturer's instructions. The glass slide spotted with 28 wells was read in a microarray reader GenePix 4000B (Molecular Devices, San Jose, CA, USA) selecting the green channel for Cy3 at the 532 nm excitation wavelength. The fluorescence standard curves and quantitation of serum samples were processed with the GraphPad Prism software (GraphPad Software, San Diego, CA, USA).

### Insulin and glucose measurements

The insulin and glucose levels were determined in serum by a certified clinical laboratory specialized in the management of animal samples (Laboratorios AIMSA, ISO 15,189).

### Molecular dynamics

For the study of molecular dynamics, we used a model of peptide VSAK generated by the I-TASSER server^57^, at the highest TM server reporter. Simulations were performed employing a series of lipid bilayer systems generated by the CHARMM-GUI server and the MARTINI 22p modified forcefield^58,59^. This forcefield allowed us the use of a polarizable water model and charged amino acids. For simulations, two different bilayers were constructed, the first one consisting of an array of 36 LPS molecules in the outer leaf and 92 molecules of DOPC in the inner leaf. The second one consisted of 92 DOPC molecules in both hemi-halves of the membrane. All simulations were performed for 3000 ns, and at the start, peptides were collocated at 30 Å above the top of the membrane. We analyzed the mean square displacement (MSD) in the lateral plane of the bilayers, as an indicator of membrane fluidity.

### Statistical analysis

All statistical tests were performed with the GraphPad Prism software. Standard uptake value SUV, %ID, and temperature data were processed with a two-way ANOVA. Insulin, glucose, and cytokine measurements were evaluated by a one-way ANOVA. Statistical significance was considered at *p* < 0.05.

## Supplementary Information

Supplementary video legends.

Supplementary Video 1.

Supplementary Information 2.

Supplementary Video 2.

Supplementary Information 3.

Supplementary Video 3.

Supplementary Information 4.

Supplementary Video 4.

Supplementary Information 5.

Supplementary Video 5.

Supplementary Information 6.

Supplementary Video 6.

Supplementary Information 7.

Supplementary Video 7.

Supplementary Information 8.

Supplementary Video 8.

Supplementary Video 9.

Supplementary Information 9.

Supplementary Video 10.

Supplementary Information 10.
