# Supplementary material for: Peptide VSAK maintains tissue glucose uptake and attenuates pro-inflammatory responses caused by LPS in an experimental model of the systemic inflammatory response syndrome: a PET study
Source: Sci Rep. 2021 Jul 20;11:14752. doi: 10.1038/s41598-021-94224-2 (PMC8292390; doi:10.1038/s41598-021-94224-2)
Supplement: Supplementary file 1 — Supplementary video legends. [file 41598_2021_94224_MOESM1_ESM.doc]

**Supplementary Materials:**

Supplementary Video S1. Systemic [^18^F]FDG uptake of a control experimental animal. Video showing the systemic [^18^F]FDG uptake taken at the end of the 70 minutes acquisition time of representative control rabbit shown in Fig. 1 injected with saline solution only.

Supplementary Video S2. Systemic [^18^F]FDG uptake of a peptide VSAK treated experimental animal. Video of the systemic [^18^F]FDG uptake taken at the end of the 70 minutes acquisition time of representative rabbit shown in Fig. 1 injected with peptide VSAK (60 µg/kg).

Supplementary Video S3. Systemic [^18^F]FDG uptake of a LPS treated experimental animal. Video of the systemic [^18^F]FDG uptake taken at the end of the 70 minutes acquisition time of representative rabbit shown in Fig. 1 injected with LPS (300 ng/kg).

Supplementary Video S4. Systemic [^18^F]FDG uptake of a LPS/peptide VSAK treated experimental animal. Video of the systemic [^18^F]FDG uptake taken at the end of the 70 minutes acquisition time of representative rabbit shown in Fig. 1 injected with LPS (300 ng/kg) and peptide VSAK (60 µg/kg).

Supplementary Video S5. Systemic [^18^F]FDG uptake of a control experimental animal. Video of the systemic [^18^F]FDG uptake taken at the end of the 90 minutes acquisition time of representative control rabbit shown in Fig. 2 injected with saline solution only.

Supplementary Video S6. Systemic [^18^F]FDG uptake of a peptide VSAK treated experimental animal. Video of the systemic [^18^F]FDG uptake taken at the end of the 90 minutes acquisition time of representative control rabbit shown in Fig. 2 injected with peptide VSAK (60 µg/kg).

Supplementary Video S7. Systemic [^18^F]FDG uptake of a LPS treated experimental animal. Video of the systemic [^18^F]FDG uptake taken at the end of the 90 minutes acquisition time of representative rabbit shown in Fig. 2 injected with LPS (450 ng/kg).

Supplementary Video S8. Systemic [^18^F]FDG uptake of a LPS/VSAK treated experimental animal. Video of the systemic [^18^F]FDG uptake taken at the end of the 90 minutes acquisition time of representative rabbit shown in Fig. 2 injected with LPS (450 ng/kg) and peptide VSAK (60 µg/kg).

Supplementary Video S9. Molecular dynamics of interactions between peptide VSAK and LPS/DOPC bilayers. Molecular dynamics video showing the intereaction of a LPS/DOPC bilayer system with peptide VSAK (from 9000 to 12000 ps).

Supplementary Video S10. Molecular dynamics of interactions between peptide VSAK and DOPC bilayers. Molecular dynamics video showing the interaction of a DOPC bilayer system with peptide VSAK (from 9000 to 12000 ps).
