# Supplementary material for: Peptide VSAK maintains tissue glucose uptake and attenuates pro-inflammatory responses caused by LPS in an experimental model of the systemic inflammatory response syndrome: a PET study
Source: Sci Rep. 2021 Jul 20;11:14752. doi: 10.1038/s41598-021-94224-2 (PMC8292390; doi:10.1038/s41598-021-94224-2)
Supplement: Supplementary file 7 — Supplementary Information 4. [file 41598_2021_94224_MOESM7_ESM.pdf]

Supplementary Video S3. Systemic [^{18}F]FDG uptake of a LPS treated experimental animal.

Peptide VSAK maintains tissue glucose uptake and attenuates pro-inflammatory responses caused by LPS in an experimental model of septic shock; a PET study.

Ismael Luna-Reyes, Eréndira G. Pérez-Hernández, Blanca Delgado-Coello, Miguel Ángel Ávila-Rodríguez, & Jaime Mas-Oliva
